# Consensus Recommendation for Mouse Models of Ocular Hypertension to Study Aqueous Humor Outflow and Its Mechanisms

**DOI:** 10.1167/iovs.63.2.12

**Published:** 2022-02-07

**Authors:** Colleen M. McDowell, Krishnakumar Kizhatil, Michael H. Elliott, Darryl R. Overby, Joseph van Batenburg-Sherwood, J. Cameron Millar, Markus H. Kuehn, Gulab Zode, Ted S. Acott, Michael G. Anderson, Sanjoy K. Bhattacharya, Jacques A. Bertrand, Terete Borras, Diane E. Bovenkamp, Lin Cheng, John Danias, Michael Lucio De Ieso, Yiqin Du, Jennifer A. Faralli, Rudolf Fuchshofer, Preethi S. Ganapathy, Haiyan Gong, Samuel Herberg, Humberto Hernandez, Peter Humphries, Simon W. M. John, Paul L. Kaufman, Kate E. Keller, Mary J. Kelley, Ruth A. Kelly, David Krizaj, Ajay Kumar, Brian C. Leonard, Raquel L. Lieberman, Paloma Liton, Yutao Liu, Katy C. Liu, Navita N. Lopez, Weiming Mao, Timur Mavlyutov, Fiona McDonnell, Gillian J. McLellan, Philip Mzyk, Andrews Nartey, Louis R. Pasquale, Gaurang C. Patel, Padmanabhan P. Pattabiraman, Donna M. Peters, Vijaykrishna Raghunathan, Ponugoti Vasantha Rao, Naga Rayana, Urmimala Raychaudhuri, Ester Reina-Torres, Ruiyi Ren, Douglas Rhee, Uttio Roy Chowdhury, John R. Samples, E. Griffen Samples, Najam Sharif, Joel S. Schuman, Val C. Sheffield, Cooper H. Stevenson, Avinash Soundararajan, Preeti Subramanian, Chenna Kesavulu Sugali, Yang Sun, Carol B. Toris, Karen Y. Torrejon, Amir Vahabikashi, Janice A. Vranka, Ting Wang, Colin E. Willoughby, Chen Xin, Hongmin Yun, Hao F. Zhang, Michael P. Fautsch, Ernst R. Tamm, Abbot F. Clark, C. Ross Ethier, W. Daniel Stamer

**Affiliations:** 1 Department of Ophthalmology and Visual Sciences, University of Wisconsin–Madison, Madison, Wisconsin, United States; 2 The Jackson Laboratory, Bar Harbor, Maine, United States; 3 University of Oklahoma Health Sciences Center, Oklahoma City, Oklahoma, United States; 4 Department of Bioengineering, Imperial College London, United Kingdom; 5 Department of Pharmacology & Neuroscience, and North Texas Eye Research Institute, University of North Texas Health Science Center, Fort Worth, Texas, United States; 6 Department of Ophthalmology and Visual Sciences and Institute for Vision Research, The University of Iowa; Center for the Prevention and Treatment of Visual Loss, Veterans Affairs Medical Center, Iowa City, Iowa, United States; 7 Ophthalmology and Biochemistry and Molecular Biology, Casey Eye Institute, Oregon Health & Science University, Portland, Oregon, United States; 8 Department of Molecular Physiology and Biophysics and Department of Ophthalmology and Visual Sciences, The University of Iowa; Center for the Prevention and Treatment of Visual Loss, Veterans Affairs Medical Center, Iowa City, Iowa, United States; 9 Bascom Palmer Eye Institute, University of Miami, Miami, Florida, United States; 10 Department of Bioengineering, Imperial College London, London, United Kingdom; 11 University of North Carolina at Chapel Hill, Chapel Hill, North Carolina, United States; 12 Brightfocus Foundation, Clarksburg, Maryland, United States; 13 Department of Ophthalmology and Visual Sciences, University of Iowa, Iowa City, Iowa, United States; 14 SUNY Downstate Health Sciences University, Brooklyn, New York, United States; 15 Department of Ophthalmology, Duke Eye Center, Duke University, Durham, North Carolina, United States; 16 Department of Ophthalmology, University of Pittsburgh, Pennsylvania, United States; 17 Department of Pathology and Laboratory Medicine, University of Wisconsin–Madison, Madison, Wisconsin, United States; 18 Institute of Human Anatomy and Embryology, University of Regensburg, Regensburg, Germany; 19 Department of Ophthalmology and Visual Sciences, SUNY Upstate Medical University, Syracuse, New York, United States; 20 Department of Ophthalmology, Boston University School of Medicine, Boston, Massachusetts, United States; 21 University of Houston–Victoria, Victoria, Texas, United States; 22 Smurfit Institute of Genetics, Trinity College Dublin, Dublin, Ireland; 23 Department of Ophthalmology, Columbia University, New York, New York, United States; 24 Casey Eye Institute, Oregon Health & Science University, Portland, Oregon, United States; 25 Department of Ophthalmology and Department of Integrative Biosciences, Oregon Health & Science University, Portland, Oregon, United States; 26 Ocular Genetics Unit, Smurfit Institute of Genetics, Trinity College Dublin, Dublin, Ireland; 27 Department of Ophthalmology, University of Utah School of Medicine, Salt Lake City, Utah, United States; 28 Department of Surgical and Radiological Sciences, University of California, Davis, Davis, California, United States; 29 Department of Chemistry and Biochemistry, Georgia Institute of Technology, Atlanta, Georgia, United States; 30 Department of Ophthalmology and Department of Pathology, Duke University, Durham, North Carolina, United States; 31 Department of Cellular Biology and Anatomy, James & Jean Culver Vision Discovery Institute, Augusta University, Augusta, Georgia, United States; 32 Duke Eye Center, Duke Health, Durham, North Carolina, United States; 33 Department of Neurobiology, University of Utah, Salt Lake City, Utah, United States; 34 Department of Ophthalmology, Indiana University School of Medicine, Indianapolis, Indiana, United States; 35 Department of Surgical Sciences and Department of Ophthalmology and Visual Sciences, University of Wisconsin–Madison, Madison, Wisconsin, United States; 36 College of Optometry, University of Houston, Houston, Texas, United States; 37 Department of Ophthalmology, Icahn School of Medicine at Mount Sinai, New York, New York, United States; 38 Ophthalmology Research, Regeneron Pharmaceuticals, Tarreytown, New York, United States; 39 Department of Ophthalmology, Duke University School of Medicine, Durham, North Carolina, United States; 40 Department of Neurobiology, University of California, Irvine, Irvine, California, United States; 41 Case Western Reserve University School of Medicine, Cleveland, Ohio, United States; 42 Department of Ophthalmology, Mayo Clinic, Rochester, Minnesota, United States; 43 Washington State University, Floyd Elson College of Medicine, Spokane, Washington, United States; 44 Western Glaucoma Foundation, Sisters, Oregon, United States; 45 Santen Inc., Emeryville, California, United States; 46 Department of Ophthalmology and Department of Physiology and Neuroscience, NYU Grossman School of Medicine, NYU Langone Health, New York University, New York, New York, United States; Departments of Biomedical Engineering and Electrical and Computer Engineering, New York University Tandon School of Engineering, Brooklyn, New York, United States; Center for Neural Science, College of Arts and Science, New York University, New York, New York, United States; 47 Department of Pediatrics and Department of Ophthalmology and Visual Sciences, University of Iowa Carver College of Medicine, Iowa City, Iowa, United States; 48 Veterans Affairs Palo Alto Health Care System, Stanford University, Palo Alto, California, United States; 49 Department of Ophthalmology and Visual Sciences, University of Nebraska Medical Center, Omaha, Nebraska, United States; Department of Ophthalmology and Vision Sciences, The Ohio State University, Columbus, Ohio, United States; 50 Glauconix Biosciences, Albany, New York, United States; 51 Cell and Developmental Biology Department, Northwestern University, Chicago, Illinois, United States; 52 Department of Ophthalmology, Casey Eye Institute, Oregon Health & Science University, Portland, Oregon, United States; 53 Genomic Medicine, Biomedical Sciences Research Institute, Ulster University, Coleraine, Northern Ireland, United Kingdom; 54 Beijing Institute of Ophthalmology, Beijing Tongren Eye Center, Beijing Tongren Hospital, Capital Medical University, Beijing, China; 55 Department of Ophthalmology, University of Pittsburgh, Pittsburgh, Pennsylvania, United States; 56 Biomedical Engineering Department, Northwestern University, Evanston, Illinois, United States; 57 University of Regensburg, Regensburg, Germany; 58 Department of Pharmacology and Neuroscience, North Texas Eye Research Institute, University of North Texas Health Science Center, Fort Worth, Texas, United States; 59 Wallace H. Coulter Department of Biomedical Engineering, Georgia Institute of Technology; Emory University School of Medicine, Emory University, Atlanta, Georgia, United States; 60 Duke Ophthalmology, Duke University, Durham, North Carolina, United States

**Keywords:** mouse model, conventional outflow, glaucoma, intraocular pressure, ocular hypertension, outflow facility

## Abstract

Due to their similarities in anatomy, physiology, and pharmacology to humans, mice are a valuable model system to study the generation and mechanisms modulating conventional outflow resistance and thus intraocular pressure. In addition, mouse models are critical for understanding the complex nature of conventional outflow homeostasis and dysfunction that results in ocular hypertension. In this review, we describe a set of minimum acceptable standards for developing, characterizing, and utilizing mouse models of open-angle ocular hypertension. We expect that this set of standard practices will increase scientific rigor when using mouse models and will better enable researchers to replicate and build upon previous findings.

Glaucoma, a complex group of optic neuropathies, is the leading cause of irreversible blindness worldwide. Elevation of intraocular pressure (IOP) is a key risk factor for the most common form of primary open-angle glaucoma (POAG). Although there are no cures for glaucoma, prospective randomized multicenter studies provide convincing evidence that pharmacological and/or surgical interventions that specifically lower IOP can slow the progression of disease in all forms of glaucoma.^1^^–^^5^ The purpose of this position paper is to provide the research community with minimum acceptable standards to develop and evaluate mouse models of ocular hypertension (OHT), as well as provide guidelines for developing and validating new mouse models of OHT.

IOP is generated by the production and drainage of aqueous humor (AH), a clear fluid that provides nutrients to the avascular tissues of the anterior eye. Under normal physiological circumstances, AH is actively secreted by the ciliary epithelium and flows between the lens and iris into the anterior chamber, where it exits via two pathways, the conventional pathway and the unconventional (uveoscleral and uveovortex) pathway. AH drainage via the conventional outflow pathway is pressure dependent; this route is comprised of the trabecular meshwork (a sponge-like connective tissue), Schlemm's canal (SC), collector channels, and the episcleral vasculature, where AH joins the venous system (Fig. 1). A smaller fraction of AH is drained passively through the unconventional pathway, where it is removed from the anterior chamber either via the uveal veins into the vortex veins or by passing through the interstitial spaces of the sclera to enter the scleral and orbital vessels.^6^ The scleral route is usually used by particles traveling in the unconventional pathway. Unconventional outflow passes through the ciliary muscle and into the suprachoroidal space, but the pathway taken from the suprachoroidal space to exit the eye has been debated.

**Figure 1.**
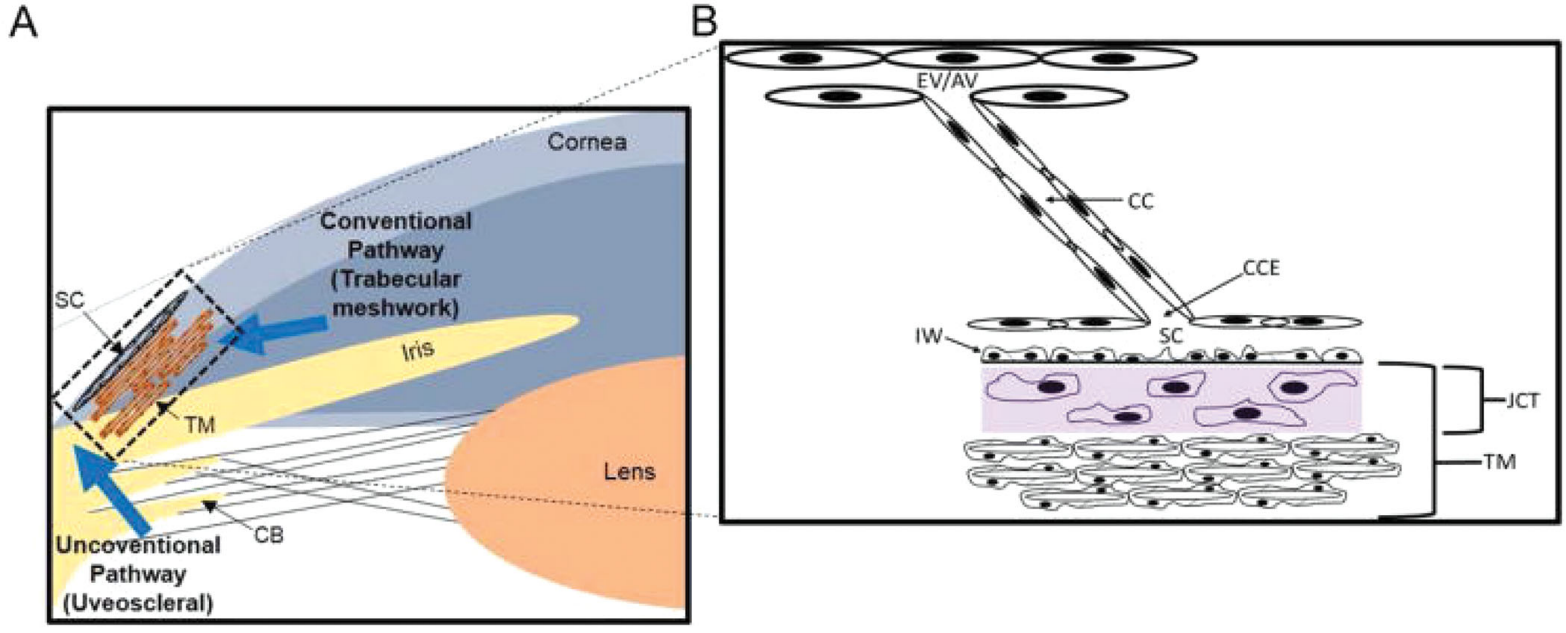
Schematic diagram of outflow pathway and structures in the trabecular meshwork. (**A**) Schematic diagram depicting conventional and uveoscleral pathway in the anterior eye chamber. (**B**) A magnified view of trabecular meshwork (TM) depicting distal regions including collector channel entrances (CCEs), collector channels (CCs), episcleral vein (EV), and aqueous vein (AV). CB, ciliary body; SC, Schlemm's canal; IW, inner wall; JCT, juxtacanalicular. Reprinted with permission from Carreon T, van der Merwe E, Fellman RL, Johnstone M, Bhattacharya SK. Aqueous outflow - a continuum from trabecular meshwork to episcleral veins. *Prog Retin Eye Res*. 2017;57:108–133.

The primary resistance to AH drainage from the anterior chamber occurs in the conventional outflow pathway. To maintain normal IOP, the interface between the trabecular meshwork (TM; juxtacanalicular region) and the inner wall of SC actively generates and modulates 50% to 75% of the total outflow resistance, whereas the remainder of resistance (25%–50%) occurs distal to the outer wall of SC, as shown in human eyes.^7^^–^^12^ In patients with POAG, an increase in fibrillar extracellular matrix deposition has been observed in the juxtacanalicular region.^13^^–^^16^ This increase correlates with axonal damage of the optic nerve.^17^ Moreover, there is increased stiffness of both the juxtacanalicular region and the SC endothelium in eyes with POAG that correlates with low outflow facility and likely contributes to elevated IOP.^13^^,^^18^ Emerging evidence suggests a role for the distal outflow vessels in regulating IOP via vasomotion. Further investigation with newly identified agents that relax vessel constriction in this area should prove useful in defining the role of distal vessel constriction in OHT and POAG.^9^^,^^19^^–^^26^ In fact, in a recent study it was shown that the effects of netarsudil, a new glaucoma treatment drug, partially involves lowering distal outflow resistance by dilating episcleral veins.^27^^–^^29^

Hereditary predispositions contribute to glaucoma risk, although the genetics are complex. To date, over 100 glaucoma and IOP-related genes have been identified. More work is needed to translate these discoveries to a molecular understanding of glaucoma pathophysiology in general and TM function in particular. Current challenges in human glaucoma genetics research include a lack of detailed understanding of genotype–phenotype relationships, a paucity of high-penetrant Mendelian variants for the disease, the fact that individually most of the discovered variants have very modest impact on the disease, and, finally, that POAG, the most common form of glaucoma, results from a complex interplay of genes that contribute to TM dysfunction and optic nerve vulnerability to degeneration. Based on the nature of the disease, it is clear that the genetic component of glaucoma is heterogeneous and includes many genes with modest effect sizes.^30^ It is thus necessary and critical to evaluate the contribution of the genes to glaucoma through the use of appropriate animal models.

Models of OHT in rats, dogs, cats, and monkeys exist, but each has its own challenges, including the availability of genetic resources and difficulty of genetic manipulation, ethical considerations, cost, and maintenance. Thus, mice have become an attractive animal model, as they are genetically similar to humans, and their ocular anatomy and physiology are comparable to those of humans, including that of the conventional outflow pathway.^31^^–^^37^ In addition, because mice can be readily genetically manipulated and there is a wide array of mouse resources and genetic tools, specific genes can be targeted to gain a better understanding of their role in disease or to potentially uncover modifier genes leading to a variety of phenotypes. Mice, therefore, provide powerful in vivo tools for studying complex diseases that are mediated by complex tissues, such as OHT, which is influenced by the tissues contributing to conventional outflow resistance.^38^^–^^41^

Several mouse models of OHT that aid our understanding of glaucomatous pathophysiology have been described previously and extensively reviewed.^41^ Although the purpose of this position paper is to provide the research community with minimum acceptable standards to develop and evaluate mouse models of OHT, these recommendations specifically apply to models in which OHT is produced through perturbations in the conventional outflow pathway leading to decreased outflow facility and elevated IOP.

## OHT Phenotypes in Models Currently in Use and Minimum Acceptable Standards

Mouse models of glaucoma currently described in the literature include both pressure-dependent and pressure-independent models, as previously extensively reviewed.^41^ Each model has its strengths and weaknesses. For example, models that utilize microbead occlusion, injection of oil or viscous agents, laser photocoagulation, or circumlimbal sutures are valuable for investigating the effect of OHT on neurodegeneration of retinal ganglion cells. Likewise, the DBA/2J mouse (an iris atophy/pigment dispersion model) is an important model system to study IOP elevation in pigmentary glaucoma. DBA/2J genes affecting melanosomal biology contribute to DBA/2J glaucoma and some human pigmentary glaucoma.^42^^–^^45^ The nee mouse model (congenital glaucoma model, *Sh3pxd2b^nee^* mutation) is a valuable resource for studying early-onset glaucoma, as well as for studying glaucomatous neurodegeneration.^43^^,^^46^^–^^48^ However, models in which OHT is developed or induced by physically blocking or sclerosing the AH outflow pathways are not necessarily appropriate to study TM biology or AH outflow pathology. Model systems that recapitulate the human condition and harbor the necessary phenotypes within the conventional outflow pathway are the most relevant and are necessary for assessing the role of the TM, SC, and distal region in generating elevated IOP and outflow dysfunction in open-angle glaucoma. Examples of these include genetic models of glaucoma (Myoc^Y437H^ mice), OHT induced by transduction of the TM with glaucoma-related genes (e.g., *MYOC*, *TGFβ2*, *GREM1*, *CTGF*, *DKK1*, *SFRP1*, *CD44*, Cre and inducible transgene models, genome editing),^49^^–^^56^ as well as glucocorticoid-induced OHT models.^14^^,^^57^^–^^61^ *Lmx1b* models also have direct relevance to both developmental and open-angle human glaucoma.^62^^–^^64^ We will use the Myoc^Y437H^ mouse as an example to describe appropriate characteristics of a pressure-dependent model system for studying OHT.

## MYOC^Y437H^ Transgenic Mice

Glaucoma is a multifactorial disease involving multiple genes that contribute to the phenotype; therefore, it is rare for a mutation in a single gene to be identified as a cause for the disease. One such gene is myocilin, which was initially identified in two families with early-onset IOP elevation resulting in the development of POAG.^65^ Subsequent studies demonstrated that myocilin mutations are found in up to 36% of juvenile open-angle glaucoma and 3% to 4% of patients with POAG.^66^ All disease-causing mutations of the gene appear to act in an autosomal dominant fashion.

Clinical studies indicate that some *MYOC* mutations result in an early-onset phenotype with comparatively high IOPs, whereas others impart less severe disease, similar to late-onset POAG.^67^ Based on the observation that patients with a mutation changing the tyrosine in position 437 to a histidine (Tyr437His) often develop very high IOP in their second or third decade of life, Zode et al.^68^ generated a transgenic mouse expressing human MYOC Tyr437His under the control of the cytomegalovirus promoter referred to as Tg-MYOC^Y437H^. The resulting mice express high levels of mutant myocilin in the trabecular meshwork and sclera but not in the neural retina, despite the use of a universal promoter. Tg-MYOC^Y437H^ mice are healthy and breed well, and, apart from the ocular phenotype, no other phenotypic effects of the transgene have been described. The eyes of these animals are normal at birth, but around the age of 4 months a decline in AH outflow facility can be detected in many eyes, although this does not yet correlate to increased IOP.^69^^–^^71^ The development of reduced outflow facility is accompanied by morphological changes in the TM, including decreased intertrabecular space, distended endoplasmic reticulum in TM cells, and a gradual decline in the TM cellular density.^68^

At around 6 months of age, outflow facility continues to decrease and a significant elevation of IOP can be detected in most mice. Between 6 and 14 months of age, outflow facility remains low, with a stable IOP elevation. These mice show a moderate rise in IOP during the day (approximately 16 mmHg in isoflurane-anesthetized Tg-MYOC^Y437H^ vs. 11.5 mmHg in control mice), and at night, when most mice display higher baseline IOPs, robust differences between MYOC^Y437H^ transgenic mice and control animals persist (around 21 mmHg vs. 15 mmHg, respectively).^68^^,^^72^ The development of elevated IOP in this model results in mild and progressive loss of RGCs and optic nerve axons. The absolute number of RGCs lost varies slightly among laboratories, presumably due to methodological differences, but typically around 20% of RGCs are lost at the age of 6 months and 40% to 50% at the age of 12 months in MYOC^Y437H^ transgenic mice compared with age-matched controls.^68^^,^^69^

The Tg-MYOC^Y437H^ mice have many advantages as a model of OHT; however, the degree of IOP elevation can vary based on the genetic background of the mouse strain selected for inducing the MYOC^Y437H^ mutation.^51^ In the initial description of the model, the investigators used transgenic mice on a mixed background of C57BL/6J and Swiss James Lambert (SJL), a cross commonly employed in the generation of transgenic animals.^68^ In contrast, some reports show a milder or no phenotype in mice backcrossed or otherwise crossed to C57BL/6 and other genetic backgrounds.^73^^–^^78^ The reason for these differences among laboratories may be related to differences in methodology, complex environmental interactions, inadvertent divergence into substrains, or the exact genetic context including contributions from SJL. Because the phenotype of this transgene appears to be dependent upon complicated genetic and/or environmental conditions that are lab dependent, Tg-MYOC^Y437H^ mice must be properly characterized before use in a study and the genetic background clearly defined.

One approach to counter the variability observed in the Tg-MYOC^Y437H^ phenotype is to maintain the animals in C57BL/6J breeders with confirmed genetic background. It is recommended to mate Tg-MYOC^Y437H^ mice on a C57BL/6J background to SJL mice to produce F1 litters to be used as experimental animals. This will result in all mice being heterozygous for all alleles, with half of their genome contributed by each strain. As a result, the probability that the dominant MYOC^Y437H^ transgene modifies IOP is maximized. Although this approach can easily be maintained, it poses difficulties when the experimental design requires elevated IOP in knockout mice, which are mostly available on a C57BL/6J background. Simply crossing these into Tg-MYOC^Y437H^ mice will result in a higher proportion of C57BL/6J and a reduced IOP phenotype that can be mistaken for a “rescue.” An appropriate breeding strategy would instead involve transfer of the knockout alleles into both C57BL/6J and SJL strains and subsequent production of experimental mice as outlined above. Although this is a slow process, it may be a viable solution for laboratories focused on studying specific genes. Investigators seeking to evaluate the effects of multiple genes in glaucoma may want to consider other models or other approaches that can account for the experimental bias.

**Summary of Recommendations**

• Tg-MYOC^Y437H^ mice have many advantages as a mouse model of OHT.
• Genetic background and other factors affect the degree of IOP elevation and rate of age-related axon loss in Tg-MYOC^Y437H^ mice. A detailed description of genetic background, breeding strategies, and housing environment/diet should be included in all publications.

## Minimum Acceptable Standards for the Description of Mouse Models of OHT

Mouse models used to study outflow physiology must demonstrate elevated IOP or at the very least decreased outflow facility and open iridocorneal angles and must include histologic descriptions of the morphology of the conventional outflow tract (TM, SC, collector channels, and intrascleral and episcleral regions). Additional features may include assessment of TM cell numbers and identification of high- and low-flow regions. Analysis of the TM ultrastructure by electron microscopy techniques is also preferred to assess extracellular matrix (ECM) changes in juxtacanalicular tissue (JCT), cellular health, cell loss, and intactness of trabecular beams, JCT, and the inner wall of SC.^34^^,^^36^^,^^57^^,^^79^^–^^82^

### Intraocular Pressure Measurements

There are several established methods to measure and analyze IOP, with different groups trusting different methods. The most common and least invasive approach to IOP measurement is with the TonoLab rebound tonometer (iCare Finland, Vantaa, Finland).^83^^–^^88^ In addition, microneedle cannulation methods also provide accurate IOP measurements; however, caution should be taken when performing repeated measurements.^89^^,^^90^ Here, we describe only best practices for measuring IOP using the TonoLab tonometer, which was specifically designed for the rodent eye. The TonoLab tonometer has been well studied, and calibration studies have demonstrated its accuracy in inbred mice and spontaneous and induced mouse models of glaucoma.^83^^,^^91^ However, changes in corneal and other ocular properties have the potential to affect readings, and appropriate caution should be taken to account for mouse strain differences or other factors that influence corneal biomechanical properties.

The TonoLab rebound tonometer uses a small force to propel a very lightweight probe against the cornea. Because the resulting impact with the cornea is slight, local corneal anesthesia is not necessary.^92^ This procedure can be done in conscious mice following acclimation to handling procedures and can even be performed several times daily by a skilled user. The animals tolerate the measurement process well when they have become acclimated to the tonometry procedure, and there are no undesirable effects on the ocular surface (if used appropriately) either during the IOP measurement or afterward.^83^ In order to measure the IOP, the user must lightly press the measurement button. The tip of the probe should contact the central cornea with a perpendicular trajectory with the probe tip positioned 1 to 4 mm from the cornea surface. Whiskers may have to be trimmed to prevent them from getting in the way of the probe. For each IOP measurement, the tonometer probe strikes the center of the cornea six times consecutively; after each successful measurement, there is a short beep. A double beep indicates an inconsistent reading, which the instrument excludes. After six successful measurements, the IOP is computed using an algorithm based on probe incident velocity and deceleration, and the resulting value is shown on the display. It is best practice to repeat the measurement series at least three times and then average the three resulting readings.

The TonoLab rebound tonometer is designed and advertised to be handheld; however, some users find it important to have the tonometer stabilized and fixed with clamps connected to a ring stand. Mounting and securing the tonometer can eliminate any slight movements by the user, especially when pressing the measurement button, resulting in more consistent measurements and fewer error messages. A foot switch can also be useful to keep the tonometer from moving during measurements. In addition, it is necessary to keep the probe in a horizontal orientation, which can be difficult without the tonometer securely mounted.

The mice should be acclimatized to the procedure at least 1 week prior to measurement. Handling of mice should be minimized and must be done gently so as not to stress the mice. Ambient noise levels should be kept as low as possible. The investigator needs to be calm and handle the mice gently, as mice are sensitive to stress, which can skew IOP measurements.

Many investigators find it best to measure IOP in anesthetized mice. It is well known that anesthesia affects IOP, and care should be taken to acknowledge and/or avoid this occurrence.^84^^,^^85^ Specifically, anesthetic agents, including xylazine, lower IOP, whereas ketamine usually appears to increase IOP^84^^,^^85^^,^^93^^,^^94^; however, some reports describe ketamine as either having no effect on IOP or reducing IOP in a time-dependent manner.^93^^,^^95^^,^^96^ Different doses, mouse strains, routes of administration, or environments may contribute to these differences. To avoid an effect of ketamine/xylazine, it is recommended that intraperitoneal injections of 99 mg/kg ketamine and 9 mg/kg xylazine be used and that measurements are made within the first 12 minutes after injection. Adequate anesthesia is usually obtained in 3 to 4 minutes.^85^ Another common and well-established method is to use isoflurane anesthesia, although it should be noted that IOP drops steadily after isoflurane, especially in the first 5 minutes of isoflurane exposure^96^^,^^97^; therefore, IOP should be measured within 2 to 3 minutes of anesthetic exposure. To alleviate the effect of anesthesia, IOP measurements can also be taken in conscious animals.^51^^,^^83^ However, this method is more challenging and great care must be made to train the animals for at least a week before measuring pressures. For conscious IOP measurements, the animals can be gently restrained in a DecapiCone, placed on a custom-made restrainer (similar to those pictured in Wang et al.^83^ or Nissirios et al.^98^), and then placed on a platform of adjustable height. Whatever the method of IOP measurement, all details should be clearly stated in the methods of publications.

Whenever possible, the investigator measuring IOP should be masked to the genotypes or experimental status of the animals, and animals of different genotypes/treatment should be interspersed during measurements of IOP. The time of day when IOP measurements are taken should be consistent throughout an experiment, as IOP changes with natural circadian rhythms.^85^^,^^99^ When IOP is measured during the night or lights-off cycle, the use of a red light is widely accepted as a practical means to minimizes disruptions to circadian patterns^100^ and thus helps to maintain normal fluctuations in IOP, which is also circadian.^101^^,^^102^

Any genetic and/or procedural methods that impact the health or biomechanical properties of the cornea may affect the ability of the tonometer to provide accurate measurements; therefore, documentation of such effects, as well as control experiments (calibrations), must be included to demonstrate accurate IOP measurements. Although suppliers guarantee calibration, calibration should be periodically manometrically checked as previously described.^91^ Finally, sex differences in IOP, although reportedly modest, have been observed in some strains of mice.^85^ Given the NIH policy on sex as a biological variable, it is important for US investigators that appropriately powered groups of male and female mice be included in IOP studies unless scientific justification is provided otherwise.

**Summary of Recommendations**

• The TonoLab rebound tonometer is an accurate, non-invasive tool to measure IOP in mice.
• The tonometer should be used in a manner that minimizes tonometer movement and allows the tip of the probe to remain horizontal and strike the center of the cornea perpendicularly.
• Repeated successful measurements are taken to get one IOP value from the TonoLab. A total of at least three resulting IOP values should be averaged per eye.
• If using isoflurane anesthesia, IOP measurements should be taken within 2 to 3 minutes of anesthetic exposure.
• If using ketamine/xylazine anesthesia, IOP measurements should be taken within 12 minutes of anesthetic exposure.
• The time of day at which IOP measurements are taken should be noted and remain consistent throughout the course of an experiment.

### Ex Vivo and In Vivo Outflow Facility Measurements

Outflow facility is typically measured by perfusion, in which fluid is either (1) infused into the eye at one or more known pressures and the resulting flow rates are measured (pressure-controlled approach), or (2) infused into the eye at one or more known flow rates and the resulting pressures are measured (flow-controlled approach). Perfusion of mouse eyes ex vivo or in vivo is a technically challenging procedure that requires excellent laboratory skills, suitable hardware, attention to minute details, and appropriate data and statistical analytical approaches. It can take 6 or more months for a researcher to become proficient at perfusion measurements in mouse eyes. Here, we describe best practices for both ex vivo and in vivo outflow facility measurements.

#### Ex Vivo Outflow Facility Measurements

Ex vivo measurements of outflow facility have the advantage of functionally isolating the conventional outflow pathway and avoiding the added difficulty of measuring/accounting for other physiological variables that are relevant during in vivo perfusion (episcleral venous pressure, aqueous inflow rate, unconventional outflow). Below we describe important considerations for conducting physiologically appropriate ex vivo mouse eye perfusions.

##### Hardware Requirements

It is of interest to consider normal AH inflow rate in the mouse eye, as this will determine accuracy and resolution of the hardware for perfusing the eye. Furthermore, perfusing eyes at flow rates far in excess of normal inflow rates can lead to non-physiological effects and must be avoided. The determination of inflow rate in the mouse eye has been described in several studies. Cole^103^ tabulated the anterior chamber turnover rate in various mammalian species as determined by various dilution methods and reported an average turnover rate, defined as inflow rate divided by anterior chamber volume, of 0.014 min^−1^, with a range of 0.009 to 0.021 min^−1^ (i.e., 0.9%–2.1% of the anterior chamber volume is turned over per minute). Aihara et al.^104^ reported a mean aqueous volume of 5.9 µL in 8- to 12-week-old Swiss white mice; however, this is probably an overestimate because total ocular volume for mice in this age range is 20 to 25 µL,^105^ and it is unlikely that AH occupies 25% to 30% of total mouse globe volume because of the large volume occupied by the crystalline lens and vitreous body. Nonetheless, by combining this anterior chamber volume with Cole's average turnover rate, the inflow rate is calculated to be 53 to 125 nL/min.

A drawback of the above approaches is that they rely on extrapolation of data from both rat and mouse, which may not be valid. Millar et al.^106^ used an experimental approach in adult male BALB/cJ mice (30–42 weeks), together with a modified Goldmann's equation, to estimate an inflow rate of 140 nL/min. Aihara and colleagues^104^ used an in vivo dilution technique in NIH Swiss White mice (8–12 weeks of age) to determine an inflow rate of 180 nL/min. Both Toris et al.^107^ and Zhang et al.^108^ used fluorophotometric approaches modified for the mouse eye to measure inflow rates, and they obtained flow rates of 90 to 200 nL/min in female CD-1 mice at least 6 months of age (depending on anesthesia) and 60 nL/min in C57BL/6 animals 4 to 6 weeks old, respectively. Toris noted that certain anesthetics depressed inflow, suggesting that these inflow rates may be underestimates, although this effect may be partially offset by the expectation that diffusion of fluorescein in the smaller mouse eye would be greater than in larger eyes, leading to an overestimate of inflow rates.

Despite the challenges of the small mouse eye, there is some consistency in the above results, with most data suggesting inflow rates between 60 and 200 nL/min. Some of this variability is almost certainly due to mouse strain and age^109^ and effects of anesthesia. Thus, we recommend avoiding perfusion of mouse eyes at flow rates in excess of these values.

Further, because of the low flow rates of AH produced by the ciliary body in mouse eyes, the resolution in flow rate determination must be better than 10 nL/min for making outflow measurements. To put this in context, at 10 nL/min, it takes several days to form just one drop of fluid. Note that this resolution requirement applies to both pressure- and flow-controlled approaches; for example, even in a flow-controlled approach, inaccuracies can be introduced by miscalibration or intermittency of the pump delivering the flow.

In addition to accurate measurement of flow, the resolution of pressure measurements should be better than 0.5 mmHg, although higher resolution is recommended. Further, all system elements (pressure transducer, flow transducer, and/or syringe pump) must be independently calibrated and demonstrate adequate resolution. Validation of a system can be demonstrated by perfusing glass capillaries of known diameter and comparing the measured flow resistance (numerical inverse of outflow facility) to the analytical estimate determined from Poiseuille's law or by independent means. Additionally, due to the sensitivity of the flow and pressure sensors required, perfusion systems should be isolated from room air movements and placed on a sturdy surface (preferably a vibration isolation table) away from sources of vibration.^110^ Finally, due to the large surface-to-volume ratio of mouse eyes, perfusions must be performed with the eye submerged in a temperature-controlled bath or in a humidified chamber. Poor maintenance of humidity can lead to significant over- or underestimates of facility.^105^^,^^111^^,^^112^ Aqueous gels or repeated saline drops to the cornea are not necessarily a sufficient solution, as evaporation of the aqueous phase of gels or drops can create an osmotically driven flow across the cornea.^6^ A potential alternative option would be to place the mouse eye on the surface of a pool of buffered saline in a humidified chamber or have saline continually applied to the cornea.^108^ Evaluation of pressure or flow tracings over a period of time would ensure that measurements are obtained at steady state.

##### Animal Housing

Environmental stresses during the housing of the living animals (e.g., low humidity, noise) affects outflow facility measured ex vivo,^112^ so housing conditions should be monitored and carefully controlled.

##### Study Design

To test drug (or other treatment) effects, paired experiments in which both eyes are perfused simultaneously are highly recommended, whenever appropriate, due to the large inter-animal range in normal outflow facility observed even in inbred mice.^110^^,^^112^ When this is not possible, studies should be adequately powered to account for this variability. The pharmacokinetics and pharmacodynamics of drug within the eye must be considered carefully in such studies. One approach is to unilaterally pretreat one eye (e.g., using drops) with the drug of interest before enucleation and facility measurement. However, for drugs that do not readily cross the cornea, this is not feasible, and the drug must be directly perfused into the enucleated eye. The introduction of drug into larger eyes (human, porcine) is typically accomplished by anterior chamber exchange,^113^ which requires two needles to be placed into the anterior chamber or some other process for mixing anterior chamber contents, allowing measurement of both baseline (pre-drug) and post-drug facilities. Importantly, anterior chamber exchange allows precise control over the drug concentration which would otherwise be diluted without an exchange due to the presence of drug-free fluid within the anterior chamber. Insertion of two needles in the small mouse eye is technically challenging but has been demonstrated^114^; however, drugs are also often infused with a single needle. Researchers must be aware that drug introduced into the mouse eye with a single needle will not be delivered immediately to the TM and, even when it reaches the TM, will be delivered at a diluted concentration that changes over time and is less than the infused concentration. Multiple (more than three) pressures/flow rates are recommended to establish a pressure–flow relationship from which outflow facility can be derived. Confounding factors such as evaporation, temperature, humidity, and vibration or noise must be considered, controlled, and/or monitored. For rodent eyes, such considerations are especially critical due to the small size and low flow rates.

##### Handling and Cannulation of Eyes

Mouse eyes are extremely fragile, thus delicate handling is required so that eyes and fluid contents are not distorted during enucleation for ex vivo perfusions. Eyes must be dissected out of the eye socket by cutting the extraocular muscles without damaging the globe and leaving at least 1 mm of optic nerve, rather than simply proptosing the eye from the socket and cutting behind the globe. The latter approach can lead to significant errors (e.g., traction on the optic nerve that causes undetectable leaks at the optic nerve head or severe deformation of the ocular contents). Remnants of orbital fat and muscle tissue should be removed to allow inspection of the sclera for any damage and ensure that they do not provide resistance to flow.

Cannulation and needle placement are also critical and are likely the most technically challenging aspects of the perfusion. For cannulation of the anterior chamber of the test eye, a finely pulled glass needle (40–100 µm) with a beveled edge is recommended, but cannulation can also be achieved with stainless steel needles.^61^ The cannulation needle should be handled by a micromanipulator to prevent damage to intraocular structures and should be visualized under a stereomicroscope to ensure proper placement (i.e., avoiding contact with the iris and lens). Orienting the needle perpendicular to the corneal surface reduces the force required during cannulation and minimizes rotation of the eye. However, this practice results in the wrong angle of entry into the relatively shallow mouse anterior chamber. Holding onto perilimbal conjunctival remnants with a forceps often helps to avoid rotation of the almost spherical mouse eye. Note that poor enucleation technique can make cannulation significantly more difficult.

##### Eye Perfusion

Placing the needle tip in the posterior chamber (PC) can avoid the problem of anterior chamber (AC) deepening that has been described in human eyes^7^^,^^115^^,^^116^ and also occurs in mice.^111^^,^^117^ In anterior chamber deepening, an adverse pressure gradient is established across the iris that leads to posterior motion of the lens and iris. This in turn deforms outflow tissues, leading the outflow facility to increase as pressure increases.^7^ However, posterior chamber cannulation is a risky technique in rodent eyes, as needle tips are typically larger than the PC, and inserting a needle often deforms or penetrates the iris and/or contacts the lens capsule. Thus, most investigators place their needle tip in the AC. AC deepening is predominantly an issue in ex vivo and postmortem perfusions where AH inflow is absent but can also occur in vivo when flow rates from the perfusion system exceed AH inflow rates.^111^ It is noteworthy that, when accounting for AC deepening in human^115^^,^^118^ and monkey^119^ eyes, outflow facility typically decreases^116^ due to collapse of the SC lumen. This collapse also occurs in mice at a clamped pressure of approximately 20 mmHg,^120^ meaning that perfusions should be conducted at pressures or flow rates that are in the physiological range and do not collapse SC. The effects of AC deepening can be partially allayed by careful data processing.

To decrease the chance of leaks, the cannulation needle can be secured to the cornea with epoxy glue. This is sometimes necessary because the mouse cornea will not always seal the area around the needle, especially if there is any rotational force (even the eye weight or fluid currents if maintained in a solution while perfused can sometimes cause these forces). Leaks are not always obvious, and they usually manifest as the IOP increases. Cyanoacrylate adhesive (e.g., Krazy Glue) should not be used, as it spreads on the eye surface, potentially occluding the episcleral vessels. If used, epoxy glue should be applied under the microscope and very sparingly around the needle entry site only.

##### Data Processing, Analysis, and Presentation

Historically, outflow facility has been assumed to be constant during a measurement period, with a corresponding linear flow–pressure relationship. Typically, assumption of a linear flow–pressure relationship has allowed calculation of facility as the slope of a flow–pressure graph, with the intercept ascribed to unconventional outflow or pressure-independent outflow. However, the flow rate at zero pressure in enucleated eyes is zero, and allowing a free intercept can lead to several-fold errors in outflow facility.^6^^,^^121^ Unfortunately, as described above, outflow facility increases with pressure in mice due to AC deepening, so assumptions of linearity should be avoided. It is important to note that *R*^2^ is a poor indicator of linearity, as it will inevitably be high for correlated parameters such as pressure and flow. A more rigorous approach is to fit a model that allows for the pressure dependence of outflow facility and then evaluate whether the nonlinearity parameter is significantly different from zero. Repeatable patterns of residuals from a linear fit are a good way to visualize nonlinearity. Alternatively, linear fits can be compared with nonlinear ones, and the one with the highest correlation in individual eyes can be used to describe the relationship with the specific perfusion setup used.

**Figure 2.**
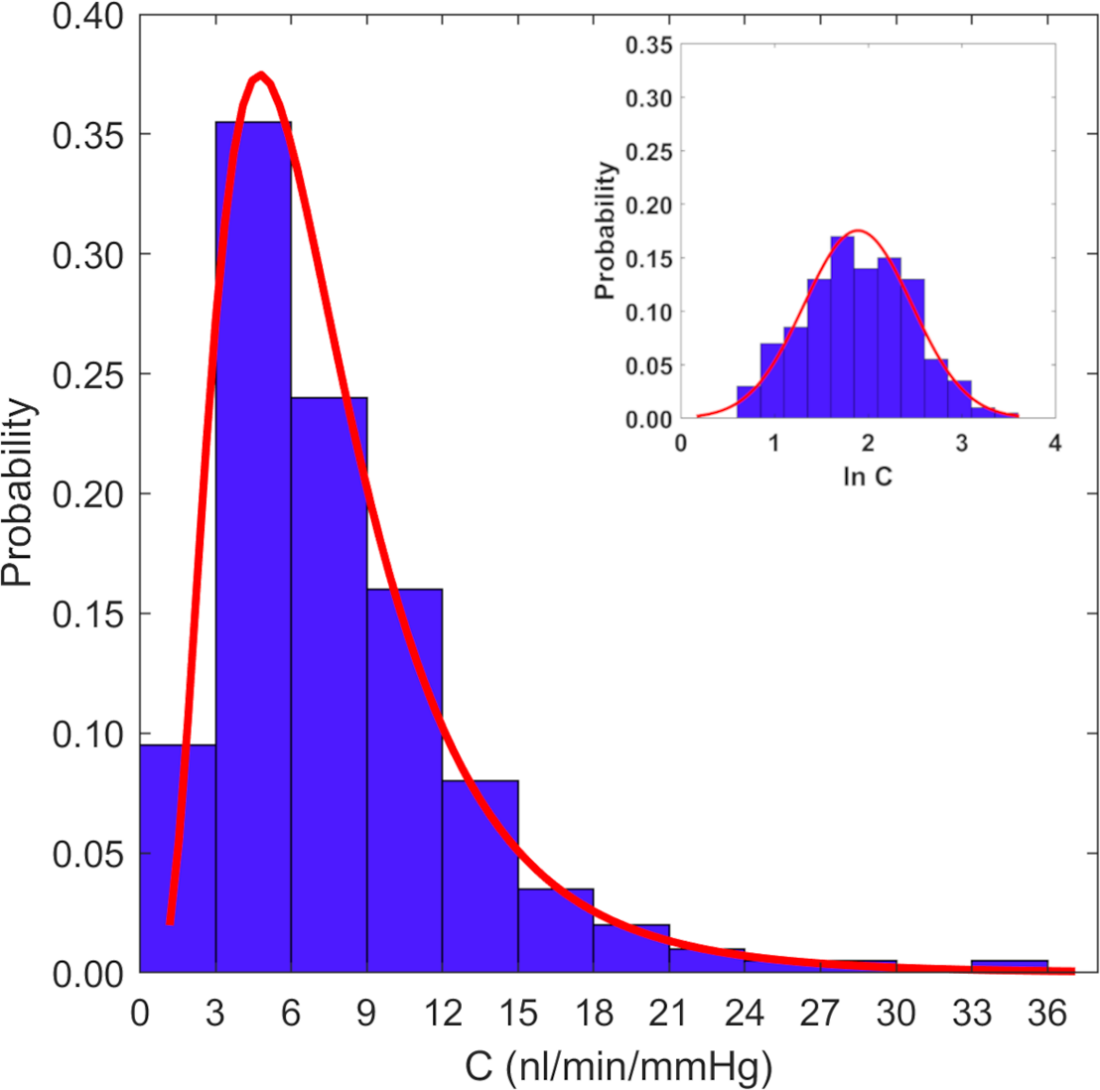
Histogram of measured facilities (C) in wild-type C57BL/6J enucleated mouse eyes. The lognormal distribution is clearly evident. The modal facility value is 3 to 6 nL/min/mmHg. Inset shows the same data, after log transformation. Reprinted with permission from Reina-Torres E, Bertrand JA, O'Callaghan J, Sherwood JM, Humphries P, Overby DR. Reduced humidity experienced by mice in vivo coincides with reduced outflow facility measured ex vivo. *Exp Eye Res*. 2019;186:107745. © 2019 Elsevier Ltd.

Ex vivo outflow facility in mouse and rat eyes is lognormally distributed when measured using the iPerfusion system (Fig. 2)^110^^,^^112^^,^^122^; that is, the distribution of outflow facility values within a population will be asymmetric and skewed toward lower values when plotted on linear axes (but symmetric when plotted on logarithmic axes. This is a consequence of the small mean outflow facility measured in mice, combined with the physical constraint that the facility must remain positive and depends on several multiplicative factors. This has three important implications.

1. Individual facility values within a given population may span a large range relative to the mean facility value. This should be considered when designing experiments or performing power calculations.
2. To account for a skewed distribution, facility values should be log-transformed where appropriate before carrying out any statistical analyses, as not doing so may violate the normality assumptions inherent in most statistical tests (e.g., Student's *t*-test) and result in erroneous statistics. For a detailed discussion of performing hypothesis testing on outflow facility values using log-normal weighted statistics, see the Supporting Information 2 document published in Sherwood et al.^110^
3. It is not appropriate to use “±” to describe the uncertainty or the spread of facility values within the population, as the lower bound will be closer to the mean than the upper bound due to the asymmetry of the log-normal distribution. Instead, we recommend using the lower and upper 95% confidence bounds to report the geometric mean, and the lower and upper values of the 2-SD range (calculated in the log domain) to report the range that encompasses 95% of the population: for example, “average outflow facility was 5.5 (4.1, 7.4) nL/min/mmHg,” and “the 2-SD range was 3.2 to 9.5 nL/min/mmHg.” When estimates of the parameters have been processed, statistical analysis must be carried out to ascertain if the observed results are statistically significant. This is typically done using hypothesis testing approaches (e.g., *t*-test, ANOVA) in the log-transformed domain.

Unfortunately, despite careful attention to detail, some perfusions fail due to a variety of reasons, including leaks, bubbles, or needle blockage. Objective exclusion criteria for selecting or rejecting perfusions should be clearly established before a study is started, and the number of rejected perfusions should be clearly reported. There are several possible reasons for exclusion:

1. Quality of the pressure–flow traces. For example, if the flow versus time plot shows large, unexpected changes over time, this may indicate a bubble or partial needle blockage.
2. Extreme (very low or very high) facility values, which can indicate leaks or needle blockage. After establishing a normative database for an individual perfusionist (Fig. 2), objective outlier detection methods can be used to remove such eyes.

It is important to establish exclusion criteria ahead of the analysis to avoid bias. Any decisions about exclusion of individual eyes should be performed in a masked fashion (without knowing the experimental group to which these eyes belong) and prior to any group comparisons.

Data presentation should focus on transparency. Rather than bar charts, plot formats that display all data points should be used (e.g., box plots with individual data points shown, beeswarm plots with a visualization of the descriptive statistics, or cello plots)^110^ that incorporate these aspects and visualize the statistical distribution upon which the statistical tests used are calculated.^123^ Standard error of the mean should be avoided; instead, 95% confidence intervals should be used to describe uncertainty on the mean, with a 2-SD range to describe the spread in the data. It is also advisable to include examples of chart recorder tracings in all publications.

#### In Vivo Outflow Facility Measurements

Measurement of outflow facility in enucleated mouse eyes as described in the previous section provides several advantages. These are centered around the concepts that outflow resistance can be studied in relative isolation, free of the complicating factors of, and potential perfusion-mediated changes in, natural aqueous secretion rate, episcleral venous pressure, tone in the ciliary muscle and iris, innervation to various structures in the anterior segment, anterior segment blood supply, and potential endocrine (and anesthesia-related) effects. However, in vivo perfusions are advantageous if one is interested in determining outflow facility (and how it may be impacted by various testing paradigms) in the living animal, which may be qualitatively and quantitatively different from the situation seen in isolated eyes perfused ex vivo, but further research is required to establish a consensus. The methodology for in vivo outflow facility measurements, lists of the equipment required to construct a constant flow infusion system, and schematics illustrating the assembly of the apparatus have been provided in detail elsewhere.^106^^,^^109^^,^^124^ Many of the same considerations described for ex vivo perfusions are also relevant to in vivo perfusions. As such, the following sections focus on issues relevant specifically to in vivo outflow measurements.

##### Anesthesia

Systemic anesthesia may^125^^,^^126^ or may not^127^^,^^128^ affect aqueous outflow facility. Both injectable cocktails and inhalation anesthesia have been utilized for in vivo studies. Of the two, the injectable cocktail approach (using ketamine and xylazine) may be the least likely to affect outflow facility in mice^127^; therefore, a mixture of ketamine (10 mg/mL) and xylazine (1 mg/mL) with a final dose of 100 mg/kg of ketamine and 10 mg/kg of xylazine is recommended as previously described.^106^ As an alternative, inhalation anesthesia can be utilized.

##### Cannulation of Eyes

As with cannulation of enucleated eyes to be perfused ex vivo, cannulation of living mouse eyes is a critical step that must be carried out with a high degree of skill. Eyes may be cannulated with needle tips placed in the AC or in the PC, as illustrated by Lopez et al.^117^ When cannulating, one must take care to place the tip of the needle at the correct starting area, immediately prior to insertion into the globe. For AC cannulation, this is against the peripheral cornea, 0.25 to 0.75 mm from the limbus. For PC cannulation, this is against the anterior portion of the sclera overlying the pars plicata, approximately 0.2 to 0.5 mm from the limbus. The needle must then be inserted in one rapid and deft motion, thereby placing the needle tip in the desired area while simultaneously angling it appropriately so as to avoid damaging internal structures.^117^ Needles must also be mounted in a suitable supporting system such that they will remain in place without movement or any kind of tractive force being exerted upon them during the course of the perfusion, which is virtually impossible (because of animal breathing) unless using a sophisticated control system.

Following successful cannulation, intracameral pressure should be adjusted using manometers to equal the eye's pre-cannulation but post-anesthesia IOP (as previously determined by tonometry). In this way, the chambers will be refilled and the eyes restored as closely as possible to their pre-cannulation state. Following this, the manometers should be switched out of the fluid perfusion circuit and the infusion pumps switched on to pump fluid at a flow rate within the physiological range.

Lopez et al.^117^ reported that, in the living mouse eye, regardless of whether the cannulating needle tip is placed in the AC or the PC, outflow resistance and hence facility are relatively independent of perfusion pressure over the perfusate flow rates of 100 to (maximally) 500 nL/min (corresponding to mean intracameral pressures of 12 to 28 mmHg). However, if perfusate flow rates are increased to 600 to 800 nL/min (very much supraphysiological in this species) then outflow resistance increases sharply. This is likely a consequence of iridiotrabecular contact or perhaps progressive collapse of the lumen of SC as flow rates increase.^120^

##### Perfusion

After commencing infusion at a flow rate of 100 nL/min, the animal should be left to equilibrate for 20 to 30 minutes. After equilibration has occurred, the infusion pump flow rates should then be increased by increments of 50 nL/min or 100 nL/min, and 10 minutes should be allowed at each equilibrated flow rate. A final flow rate of 500 nL/min should not be exceeded, for reasons described above. Following perfusion, the needles should be removed rapidly from each eye, and a return of the pressure to zero should be noted to ensure that needle blockage or leakage has not occurred. Data should be discarded from all eyes in which such a blockage has occurred.

##### Study Design

Intra-animal variation in facility (OD vs. OS as measured in naïve animals) is statistically insignificant in non-diseased normal animals.^106^^,^^109^ For this reason, where appropriate, paired study designs can be used where one eye is subjected to treatment and the contralateral eye serves as a control.

##### Data Recording and Processing

**Figure 3.**
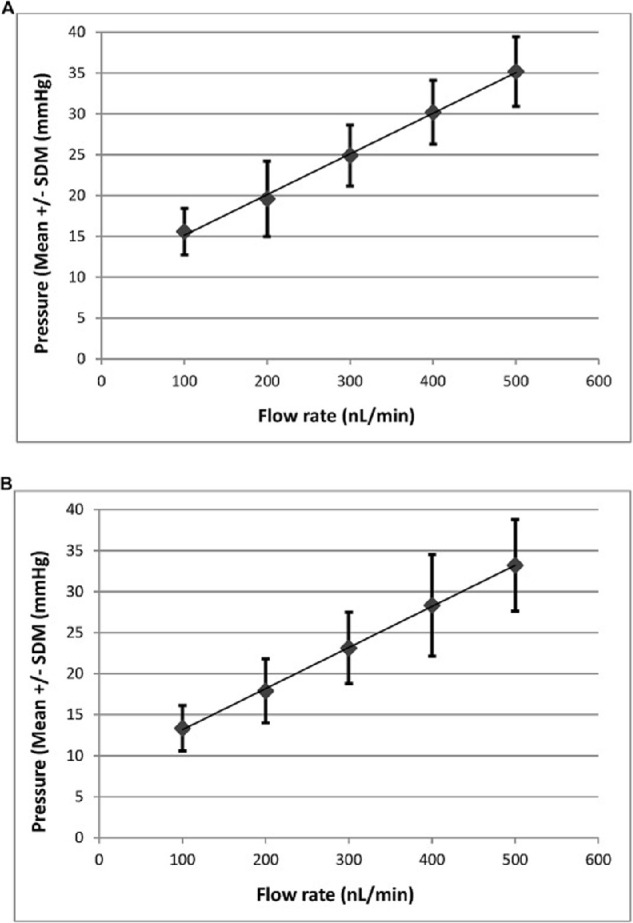
Linearity of pressure–flow rate curve in live mice. (**A**) C57BL/6J mouse pressure–flow rate curve (*N* = 6 eyes in situ in live animals; AC perfusion). Over the flow rate range of 100 to 500 nL/min, corresponding to a mean pressure of 15.58 ± 2.83 to 35.18 ± 4.26 mmHg (mean ± square deviation from the mean [SDM]), the curve approached linearity: *r*^2^ = 0.9891 ± 0.0076 (mean ± SDM); AIC (two-level linear nested design) = 128.2; AIC (three-level linear nested design) = 130. Computed facility = 19.5 ± 0.8 nL/min/mmHg (mean ± SEM). (**B**) C57BL/6J mouse pressure–flow rate curve (*N* = 6 eyes in situ in live animals; PC perfusion). Over the flow rate range of 100 to 500 nL/min, corresponding to a mean pressure of 13.36 ± 2.77 to 33.21 ± 5.57 mmHg (mean ± SDM), the curve approached linearity: *r*^2^ = 0.9882 ± 0.0032 (mean ± SDM); AIC (two-level linear nested design) = 156.2; AIC (three-level linear nested design) = 155.9. Computed facility = 21.0 ± 2.1 nL/min/mmHg (mean ± SEM). Reprinted with permission from Lopez NN, Patel GC, Raychaudhuri U, et al. Anterior chamber perfusion versus posterior chamber perfusion does not influence measurement of aqueous outflow facility in living mice by constant flow infusion. *Exp Eye Res*. 2017;164:95–108. © 2017 Elsevier Ltd.

For transparency of data expression, as with enucleated eyes perfused ex vivo, each individual data point should be shown, in the form of a scatterplot, and 95% confidence intervals should be indicated, along with one or two standard deviations from the mean. Examples of chart recorder tracings should be included in all publications. The data typically approximate a linear fit; thus, a linear regression line may be calculated. To test for approximation to linearity, the Akaike information criteria (AIC) or other appropriate test should be used^117^ (Fig. 3). If the data points at a greater than 400-nL/min flow rate do not follow the linear trend but instead indicate a sudden sharp increase in outflow resistance (as evidenced by higher than expected pressures), then these points can be eliminated from the analysis, as they are likely indicative of collapse of the lumen of SC at these relatively (and unphysiologically) high flow rates. When SC collapses, accurate measurements of outflow facility are compromised, and only preceding data points can be used for analysis.

Enucleated mouse eyes perfused ex vivo may exhibit an asymmetric distribution in individual facility values—that is, a lognormal distribution (due to the nature multiplicative nature of facility skewed toward lower values). However, no evidence for this in eyes perfused in situ in the live animal has been found,^106^^,^^109^ and data reported are not statistically significant from a normal distribution, such as Anderson–Darling, Kolmogorov–Smirnov, Ryan–Joiner (Minitab), or Shapiro–Wilk (SigmaPlot). Parametric statistical tests (such as Student's *t*-test, following a test of variance where two groups are compared, or analysis of variance where multiple groups are compared) for data analysis have been reported in live animal facility measurements, without the need for log transformation.

##### Data Exclusion Criteria

Measurement of facility in living mice is difficult and demands a great deal of care, patience, and practice. A proportion of eyes will fail for reasons such as leakage, reduction of the lumen of SC sometimes seen at lower flow rates, bleeding into the anterior chamber, or blockage of the perfusion needle. Each of these events can be identified by examination of the flow rate versus pressure graphs or after withdrawal of the needles from the eyes at the conclusion of the perfusion. Data from these eyes must be excluded. Insertion of needles into the eyes of living animals may also lead to significant breakdown of the blood–aqueous barrier, with concomitant production of plasmoid (or secondary) AH.^129^^,^^130^ However, with care, practice, and perseverance, it is possible to obtain reasonable and reproducible estimates of facility, which can show reproducible drug- or gene-induced changes in the animal.

**Summary of Recommendations**

• Outflow facility must be characterized when a new model of OHT is introduced if it is claimed that the model has a phenotype affecting the conventional outflow pathway. If outflow has not been measured in the model, it should be explicitly stated that the location of dysfunction that mediates the OHT is unknown and that any conclusions based on the assumption of a facility phenotype are therefore speculative.
• When testing the effects of agents (e.g., drugs, viruses) on outflow facility, paired experiments are strongly encouraged whenever feasible, with both eyes perfused simultaneously at pressures or flow rates in the physiological range.
• When analyzing facility data, normality should be checked. When data are found to be statistically different from a normal distribution, outflow facility values should be suitably transformed (e.g., log-transformed) and rechecked for normality before statistical analyses are carried out. Alternatively, non-parametric statistics could be applied.
• The linearity (or nonlinearity) of the flow–pressure relationship should be quantified; that is, the potential for pressure dependence of outflow facility must be considered. If flow–pressure plots are linear, facility can be determined by the slope of the linear fit of flow on pressure only if the intercept is physiologically meaningful. If they are nonlinear, an appropriate model (e.g., power law) should be used to fit the data.
• Publications that present perfusion data are strongly encouraged to include the following information as appropriate:
  ∘ Details on age, strain, and sex of the mice, along with any special diet or housing or husbandry conditions, per Animal Research: Reporting In Vivo Experiments (ARRIVE) guidelines^131^
  ∘ For ex vivo perfusion studies, details of enucleation technique, postmortem and post-enucleation times, and storage conditions of the eye prior to perfusion
  ∘ For in vivo perfusion studies, details of anesthetic used (i.e., name, dosage, frequency)
  ∘ Representative calibration data (for flow and pressure sensors and, if possible, for a “model eye”), as supplemental material
• All animals being compared should be treated and housed under similar conditions; for example, potential confounding factors could include differences in evaporation, temperature, humidity, and vibration/noise.
• All data points should be shown using suitable plot formats with inclusion and exclusion data criteria specifically described in methods, along with a statement on the number of rejected cases. Mean facility and lower and upper 95% confidence bounds should be determined and reported.

### Histological Examination

The anatomy and physiology of the conventional outflow pathway of mice have considerable similarity to that of humans. Like in the human eye, the mouse outflow tract consists of TM, JCT, SC, collector channels, and episcleral veins.^34^ However, there are some distinct differences between the two species. In the human eye, the TM is a porous and lamellated structure consisting of nine to 18 trabecular beams.^133^ Each beam has a fibrillar ECM core consisting of collagenous and elastic fibers. The core is covered by a single continuous layer of flat TM cells that rest on a complete basal lamina.^132^^–^^135^ The TM elastic fibers have a core consisting of elastin that is surrounded by a sheath that contains collagen type VI. The JCT is a loose connective tissue in which mesenchymal cells are surrounded by the ECM. Characteristic is a layer of elastic fibers (cribriform plexus) forming a fibrous network that stretches underneath the endothelial lining of SC. The elastic fibers of the plexus show the same structural characteristics as those in the trabecular beams. Connecting fibrils emerge from the cribriform plexus and form cell matrix contacts with the inner wall endothelium of SC.^132^^,^^136^ Endothelial cells of the SC inner wall rest on an incomplete basal lamina (consisting of a lamina rara and densa); considerable areas of their basal cell membrane are not supported by ECM but rather are in direct contact with the open spaces of the JCT.^136^ In the mouse eye, there are two to five layers of TM lamellae.^34^^,^^137^ As in the human TM, the lamellae contain elastic fibers that are posteriorly in contact with several longitudinally oriented smooth muscle cells which form the mouse ciliary muscle.^36^ The TM elastic fibers are not surrounded by a well-defined sheath as in the human eye. A scleral spur like in the human eye is absent. The mouse JCT is usually 1 to 2 µm wide and contains only very sparse fibrillar extracellular matrix, mostly in the form of fine fibrillar material.^53^^,^^57^^,^^79^^,^^137^^,^^138^ Most of the fibers in this material have a diameter of about 6 to 10 nm. The fibrils form typical cell-matrix contacts with SC cells. In addition, 40-nm fibers with the typical striation of collagen are observed. All types of fibers are more numerous in the posterior parts of the TM. A well-structured elastic fiber plexus that connects with the SC, as in the human JCT, is absent in the mouse eye. Some authors refer to the fine fibrillar material in the mouse JCT as “basement membrane,” a term that is appropriate as long as it is not confused with the term “basal lamina.” SC cells in the mouse eye appear not to be supported by a basal lamina (neither complete nor incomplete). Still, immunohistochemical data show patchy and discontinuous staining for collagen type IV, which is a typical basal lamina component.^57^

**Figure 4.**
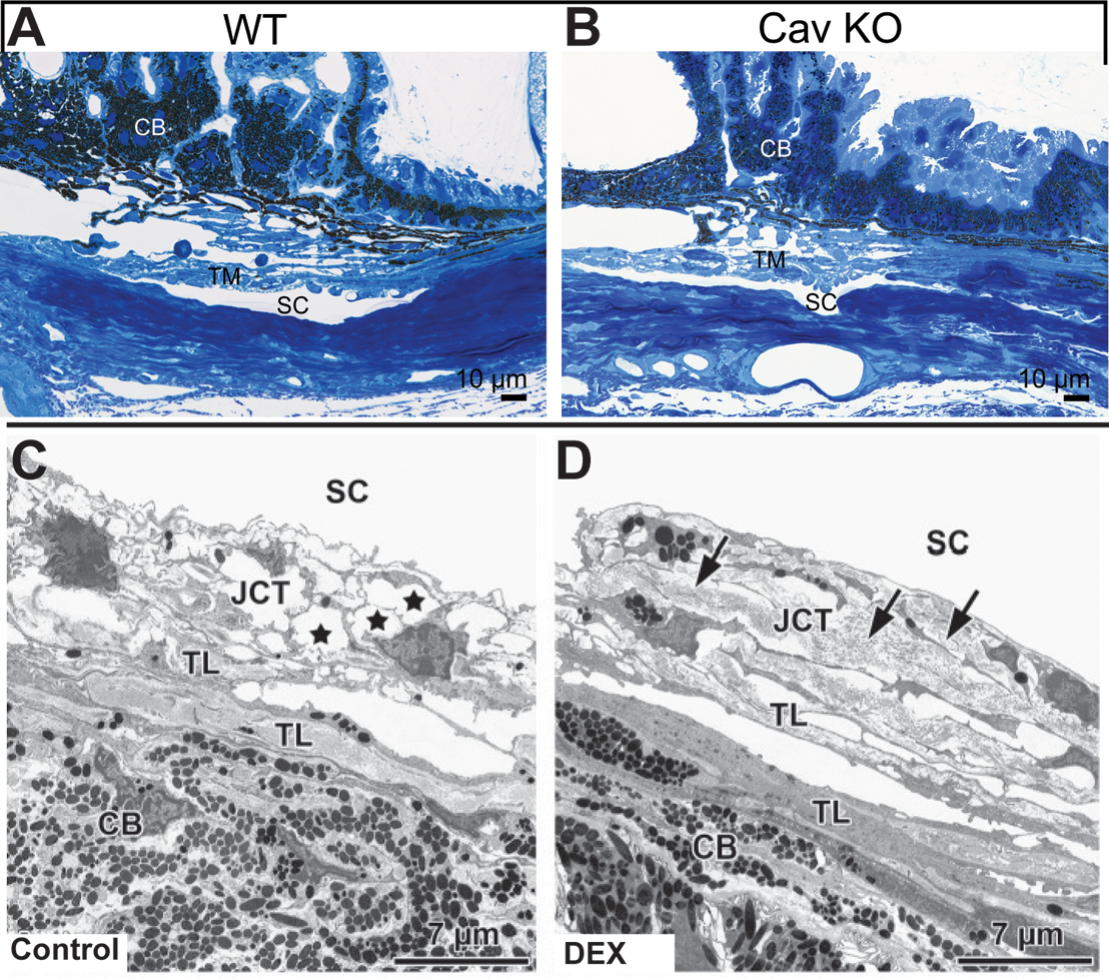
Examples of appropriate histological examination of the outflow pathway. (**A**, **B**) Semithin sections (Richardson's stain) through the iridocorneal angle of representative control and Cav-1 knockout (KO) eyes. The chamber angle is open in control and Cav-1 KO eyes, and obvious abnormalities of the CB, TM, and SC are absent. (**C**, **D**) Ultrastructural changes in the JCT region of mice treated with or without dexamethasone (DEX) for 3 to 4 weeks. (**C**) In sham-treated control mice without DEX, optically open spaces (*stars*) were often observed between JCT cells with processes extending in many directions. (**D**) In DEX-treated mice, the JCT was often filled with fine fibrillar material (*arrows*), and the JCT cells appeared elongated. TL, trabecular lamellae. **A** and **B** are reprinted from Elliott MH, Ashpole NE, Gu X, et al. Caveolin-1 modulates intraocular pressure: implications for caveolae mechanoprotection in glaucoma. *Sci Rep*. 2016;6:37127. **C** and **D** are reprinted with permission from Overby DR, Bertrand J, Tektas OY, et al. Ultrastructural changes associated with dexamethasone-induced ocular hypertension in mice. *Invest Ophthalmol Vis Sci*. 2014;55:4922–4933. © 2014 Association for Research in Vision and Ophthalmology.

For an overall assessment of mouse ocular structures, light microscopy following hematoxylin and eosin staining of paraffin or plastic sections is a reliable method. In this approach, eyes are either immersion or perfusion fixed by appropriate fixative prior to being processed and embedded. Still, immersion fixation without opening the eye results in poor fixation of the inner eye. On the other hand, when the eye is opened before immersion fixation, the anterior chamber tends to collapse, a scenario that makes it difficult to judge if the chamber angle is open and/or to exclude that anterior synechiae are present. To avoid this problem while obtaining structural preservation that is appropriate for light microscopical investigation, eyes may be opened after 30 minutes in fixative with a little cut penetrating the cornea. This time allows for hardening of the cornea/sclera and minimizes the problem of generating artifacts by mechanical distortion while cutting the cornea. While cutting and after it is very important to keep the eye submerged in the fixative to avoid air entering the eye via the cut. Any air bubbles in the eye will invariably lead to poor fixation results. Perfusion fixation has the distinct advantage over immersion in that it will much better preserve the geometry of the anterior chamber and outflow pathways. For paraffin or plastic embedded tissue, sections are cut and placed on charged glass slides to facilitate the tissue remaining on the slide throughout the staining process. Both procedures provide adequate morphological assessment, although the ability to use semi-thin plastic sections will enable analysis of finer details, as shown in Figure 4.^36^ Paraffin sections are usually considerably thicker than plastic sections (5–12 µm vs. 1–3 µm, depending on the respective laboratory), have lower resolution, and are likely to shrink or be squeezed. Therefore, great care needs to be taken to be appropriate for TM analysis. At a minimum, sections from at least two or three different locations from three different animals should be analyzed.

Although light microscopy can provide a general morphological assessment of the entire outflow pathway, it does not allow intricate detection of the ECM and analysis of cell ultrastructure. It is therefore recommended that electron microscopy studies be performed to assess the ultrastructure of the conventional outflow pathway, as shown in Figure 4. Several studies have clearly demonstrated these techniques and described the methodology in detail.^34^^,^^57^^,^^79^^–^^82^ In short, mouse anterior segments should be embedded in plastic resin and sections cut through iridocorneal tissues using an ultramicrotome, followed by staining of the section with uranyl acetate/lead citrate. If possible, ultrastructural analysis should be done in a masked manner. The analysis will enable a masked observer to examine, for example, subcellular details of the JCT and TM, the presence of fine fibrillar material or open spaces between in the TM or JCT, the thickness of fine fibrillar material surrounding elastin fibers, and the amounts and nature of basement membrane material. To quantify any changes in the amounts of fibrillar ECM, fractions of JCT and/or SC inner wall length exhibiting ECM should be measured. For specific structures, magnification should be high enough to be able to distinguish the structure of interest, such as cell organelles (endoplasmic reticulum and mitochondria),^139^ 20-µm tracer distribution,^82^ and basement membrane of the inner wall of SC.^57^ A good example is a study in which mouse eyes were studied that had been treated with dexamethasone, resulting in a considerable increase in fibrillar ECM in the JCT.^57^ To quantify the changes in basement membrane material, fractions of SC inner wall length exhibiting continuous basement membrane material should be measured in contiguous sagittal sections spanning the entire anterior-to-posterior length of SC.^57^ The length of each continuous portion of basement membrane is summed to calculate the total basement membrane length for that section, and the total basement membrane length is divided by the total inner wall length for that section computed. A comparable protocol should be used when studying JCT ECM, albeit the specific nature of ECM changes may require adjustments of the protocol. Because there are clear differences in the areas of the outflow tract (i.e., high flow vs. low flow regions)^82^ an analysis of the statistical power is strongly recommended. Several sections from each quadrant from at least three different animals are necessary. In addition, when looking for drug-induced changes, the perfusion of tracer prior to fixation may be very helpful to examine high- versus low-flow regions. Scale bars should be included for all images.

**Summary of Recommendations for Histological Examinations**

• Assessment of the iridocorneal angle should be possible and preferred when a new model is evaluated.
• Stained paraffin or semi-thin plastic sections are acceptable if of good quality to assess the general morphology of the angle structures and the conventional outflow pathway.
• Electron microscopy is necessary to analyze ultrastructure of the conventional outflow pathway.

### General Recommendations for Proper Mouse Handling and Experimental Design

#### Nomenclature: Proper Strain and Mutation Descriptions for Mice

It is important and essential to rigorously maintain and report mouse strain information to improve reproducibility. Laboratory mice originate from a variety of sources. Most mice have contributions from both *Mus musculus musculus* and *Mus musculus domesticus.* Therefore, mice should not be referred to by species name but by their specific nomenclature. Inbred strains that are produced in the lab have defined backgrounds and thus require nomenclature conventions.

A parental inbred strain will have a designation made up of uppercase, Roman letters or a combination of letters and numbers (e.g., C57BL, DBA, 129) followed by a forward slash and a Laboratory Registration Code of the institution that maintains the strain. In addition, substrains will have the substrain symbol and Laboratory Registration Code; for example, in C57BL/6J, the J stands for The Jackson Laboratory, or JAX. The most commonly used strains have standard abbreviations (e.g., C57BL/6J = B6, 129S1/SvlmJ = 129S). Hybrid strains of the first generation (F1) are named with female parent listed first followed by male parent (e.g., B6129SF1/J derived from a B6 female mated to a 129S male).

For mutant alleles, the unique identifiers include genetic background, relevant gene/allele name, technology used to generate the mouse (targeted mutation or “tm,” transgenes or “Tg,” induced and spontaneous mutations), the research group that generated the mouse, and the institution maintaining the strain. An example of nomenclature for a targeted mutation, flox, or knockout is B6.129P-*Tcrb*^tm1Mom^/J, where *Tcrb* is the gene, tm1 is the targeted mutation, and Mom is creator lab code. An example of transgene nomenclature is B6; Cg-Tg(PDGF-APP)5Lms/J, where Cg stands for congenic, Tg for transgenic, and PDGF-APP is promoter gene, founder line, creator lab code. More detailed description of mouse nomenclature can be found at the following websites (accurate at the time of publication):

1. Mouse Genome Informatics (MGI) guidelines for nomenclature of genes, genetic markers, alleles, and mutations in mouse and rat (http://www.informatics.jax.org/mgihome/nomen/gene.shtml#gkomp)
2. Top seven tips for understanding mouse nomenclature (https://www.jax.org/news-and-insights/jax-blog/2013/august/top-seven-tips-for-understanding-mou-se-nomenclature)

#### Genetic Factors

A key factor in the success of an experiment is a well-managed mouse colony, which is critical for maintaining genetic stability and reproducibility. Some general tips for good colony management include the following: (1) A well-defined breeding rotation and mating scheme should be in place so that one can avoid selecting extreme phenotypes (mild or strong). (2) A diligent record of pedigree should be kept so that any phenotypic deviants can be removed. (3) Genetic backgrounds should be refreshed regularly by backcrossing mice every five to 10 generations to the appropriate strain. (4) Cryopreserve unique strains so mice with the original genotype and phenotype can be recovered.

Understanding the influence of a genetic background on experimental outcomes is critical to interpreting experimental results. When the genetic mutation is the only variable, equal numbers of male and female age-matched littermates of each genotype (wild-type, homozygous mutant, and heterozygous) should be used. In genetic studies, control littermates are typically available. In the event that they are not, controls of ideally the same background strain (and raised under similar conditions) may be used. However, it must be remembered that when a mutation is crossed from one strain to another then genes closely linked to the mutation and that differ between the strains can confound interpretations and be systematically different between wild-type and mutant littermates.^38^ This is less of an issue after backcrossing, as the linked interval will decrease in size. Nevertheless, the possible impact of linked genes should be considered. Given that IOP can vary broadly between strains,^85^ a clear understanding of the range of IOP values and also outflow facility within the base strain of any genetic background is critical for the interpretation of the experimental data from mutant mice of the same background.

#### Environmental Factors and Housing Conditions

Mice should be group housed and the cages changed at least once weekly. If any cage appears soiled between scheduled changes, the mice should be placed in a clean cage. When possible, mice should be acclimated to the procedure room for at least 1 week prior to measurement (i.e., for IOP measurement practice). Mice are generally maintained on a 14-hour light/10-hour dark cycle or 12-hour light/12-hour dark cycle. It is critical that researchers and technicians do not enter the mouse room or turn on lights during the dark cycle. The mice are generally maintained at temperatures of 65°F to 75°F (∼18°C–23°C) at 40% to 60% humidity. A consistent diet, with fat content typically ranging from 4% to 11%, should be fed, and water should be accessible at all times.

Mice should be handled gently and as little as possible, especially when females are pregnant or have new litters. It is critical to minimize noise and vibration, as these can cause stress, reduce breeding performance, and affect physiologic parameters such as IOP. Perfumes and other strong odors that could reduce breeding performance and induce stress should be reduced. Using gloves and forceps can minimize scent cues. Enrichment using Nestlets (Animal Specialties and Provision, Quakertown, PA, USA), NestPaks (W.F. Fisher and Son, Branchburg, NJ, USA), and Shepherd Shacks (Shepherd Specialty Papers, Richland, MI, USA) can help alleviate stress and improve breeding for some strains.

#### Powering a Mouse Study

The variability of the outcome measures in both control and experimental groups will dictate the numbers of mice needed. Several pieces of information are required for sample size calculations, including (1) difference in the respective outcome measures between groups to be detected (or frequency of the outcome for each genotype/treatment arm); (2) variation in the outcome for each group (these may be different); and (3) how many follow-up time points will be included. Many of these inputs are from pilot studies followed by rigorous statistical analysis (*P* value, effect size, and power) or from the literature. If one has not found significance based on a priori sample size calculations, then the initial inputs to the power calculation can be revisited. Was there more variation than expected? Is the difference observed clinically meaningful? Additionally, some mice may become ill, may die, or may be lost to equipment failures before completing a study. Therefore, it is wise to include a few extra mice in each study cohort beyond the calculated sample size from a pilot study and power analysis. Adding mice to an experiment that is not demonstrating meaningful differences is unlikely to achieve a robust outcome. Finally, the reality of intrastrain variation should not be overlooked.^140^ Wild-type mice of the same strain may be used as controls for experiments, and if these mice are not littermates then intrastrain variation resulting from genetic drift may influence outcomes, as can cage and litter effects. Responsible reporting of methods in publications is critical for reproducibility in mouse research. Key details that should be included in publications are descriptions of mouse age, sex, and number; complete nomenclature; housing conditions; and appropriate statistical analyses.

**Overall Summary**

• Models should meet the minimum standards for OHT or outflow facility phenotypes as listed in each section above.
• Selection of a glaucoma mouse model depends on the hypothesis to be tested.
• All models have specific advantages and limitations.
• Animal handling has a direct impact on outcomes.
• Be aware of mouse strain differences.
• Available genetic and phenotyping tools make the mouse a powerful model.
• Use caution when generalizing mouse results to human glaucoma.
